# Effectiveness of wireless emergency alerts for social distancing against COVID-19 in Korea

**DOI:** 10.1038/s41598-022-06575-z

**Published:** 2022-02-16

**Authors:** Dahye Yeon, Myunghwan Kwak, Ji-Bum Chung

**Affiliations:** School of Urban and Environmental Engineering, Ulsan National Institute of Science and Technology (UNIST), UNIST-Gil 50, Ulsan, 689-798 Republic of Korea

**Keywords:** Diseases, Health care

## Abstract

This study aimed to evaluate the effectiveness of wireless emergency alerts (WEAs) on social distancing policy. The Republic of Korea has been providing information to the public through WEAs using mobile phones. This study used five data sets: WEA messages, news articles including the keyword “COVID-19,” the number of confirmed COVID-19 patients, public foot traffic data, and the government’s social distancing level. The WEAs were classified into two topics—“warning” and “guidance”—using a random forest model. The results of the correlation analysis and further detailed analysis confirmed that the “warning” WEA topic and number of news articles significantly affected public foot traffic. However, the “guidance” topic was not significantly associated with public foot traffic. In general, the Korean government’s WEAs were effective at encouraging the public to follow social distance recommendations during the COVID-19 pandemic. In particular, the “warning” WEA topic, by providing information about the relative risk directly concerning the recipients, was significantly more effective than the “guidance” topic.

## Introduction

Since 2020, the coronavirus disease 2019 (COVID-19) has spread worldwide and caused many deaths. The Republic of Korea experienced a surge in the number of confirmed cases at the initial stage of the pandemic. Since the first outbreak, the Korean government has implemented various policies to flatten the COVID-19 curve^1^. The Korean government stressed that its policies were based on transparency, openness, civic engagement, and innovativeness. They announced “a strategy of robust and pre-emptive testing, tracing, and treatment” to prevent the spread of COVID-19 without lockdown in the country and cities^2^. Based on the previous experience of infectious diseases, such as the Middle East Respiratory Syndrome, the Korean government conducted extensive epidemiological investigations. Furthermore, they used information and communications technology such as wireless emergency alerts (WEAs) and websites to share information effectively^1^.

Government policies and active civic engagement are important factors for effective infectious disease prevention and control. In this regard, the disease control authorities need to use effective risk communication tools to encourage public preventive measures. WEAs via mobile phone, a representative measure providing disaster information, have been widely used for delivering COVID-19 warnings and encouraging social distancing in Korea. Generally, mobile phones are readily accessible to the owners and can deliver messages everywhere^3^. Therefore, WEAs via mobile phones have the advantage of easy and quick accessibility. However, the number of letters in WEA messages is limited; hence, the messages are succinct. Another more general platform that people use to obtain information is news media. Appropriate news media attention can increase risk perception, decrease public confusion in an emergency^4,5^, and enhance the effects of social distancing^6^.

In Korea, the WEA service based on cell broadcast service (CBS) technology has been implemented since 2005, and local governments and central authorities have had the power to send such messages since 2017^7^. They send warning messages to individual cell phones connected to the base stations of mobile service providers in a designated area. These messages are short and include emergency alerts and guidance for emergency response^1^. As a broadcasting service, CBS sends a message once and does not require phone numbers, unlike the short message service^8^. The Korean central authorities and local governments issued warning messages about various hazards, including typhoons, fires, fine dust, and infectious diseases. Since 2020, WEA messages have increased dramatically due to COVID-19, amounting to nearly 50 times more than the previous year. Therefore, some media have reported that the excessive use of WEA could cause fatigue in the Korean public.

This study aimed to analyze the effect of WEA messages on the public behaviors in Korea, where WEAs have been used most actively. We attempted to measure citizens’ participation in social distancing policies, such as staying at home, indirectly using weekly public foot traffic data. Public mobility and activity are closely related to the spread of COVID-19^9^. Foot traffic data such as Apple and Google mobility data are good resources for measuring public mobility^10,11^. In this study, we focused on the contents of WEA messages and assessed the effectiveness of WEAs by the level of public self-restraint in outdoor movements during the COVID-19 pandemic. To understand the effectiveness of WEA messages and compare it with that of news media, we analyzed the relationship among public foot traffic, the number of WEA messages, and the number of news articles about COVID-19. Restrained traffic can be regarded as the public’s efforts to participate in social distancing policies.

**Figure 1:**
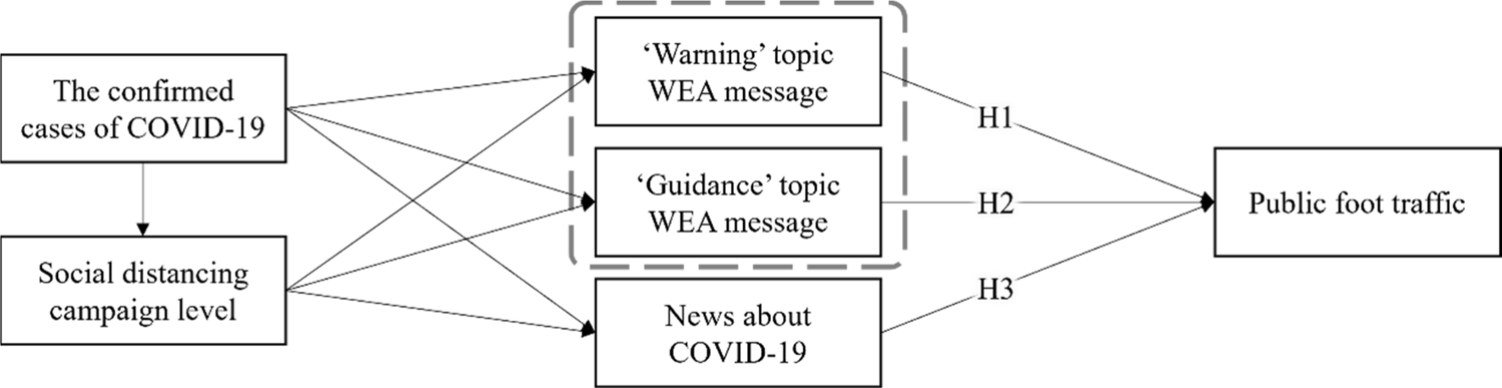
Path model with hypotheses for public foot traffic.

This study verified the following problems. First, the relationship between news media (number of news articles on COVID-19) and social distancing behavior (foot traffic data) was examined. Second, the effect of WEA’s “warning” message on decreasing public foot traffic was investigated. Lastly, the impact of the WEA’s “guidance” message on decreasing public foot traffic was examined. Figure 1 shows the conceptual model of this study. In this conceptual model, when confirmed cases of COVID-19 increase, government authorities send more WEA messages, the social distancing campaign level is higher, and the media publish more articles about COVID-19. This eventually affects the behavior of the public. Consequently, the following three hypotheses were formulated:

H1: WEA “warning” messages in a designated region negatively affect public foot traffic.
H2: WEA “guidance” messages in a designated region negatively affect public foot traffic.
H3: The number of news articles about COVID-19 negatively affects public foot traffic.

## Literature review

People’s protective actions from disaster warning can be classified into two phases based on previous studies^12,13^: warning acceptance and risk assessment. The warning acceptance phase includes receiving, understanding, and believing received warning messages. The risk assessment phase includes risk identification, risk personalization, and decision making. After these two phases, people are affected by the warning messages. If the messages are not properly addressed in these phases, WEAs do not work effectively. Their effectiveness can also be affected by several factors, such as the characteristics of the messages and receivers and credibility of the senders^14^. Furthermore, the technology itself may also limit successful protective actions, as it is not accessible to children and unfamiliar to older adults, and its process is uncertain^12^. If a false alarm is issued frequently, it will lead to warning fatigue based on the *cry wolf syndrome*^15^ or normalcy bias^16^. It decreases the government’s credibility^17^. Thus, the government should be cautious about sending messages to the public and explain the reasons for the inappropriate warning messages if a false alarm is sent^13,18^.

Several studies have measured how WEAs affect the behavior of the receivers. The number of people following the guidance is measured by observing the traffic volume and counting the number of people in shelters after receiving the warning messages^13^ or checking the receiver’s reactions to WEA messages using experimental simulations^19^. For COVID-19 cases, a study assessed the effectiveness of WEA by comparing the number of newly confirmed cases in the relevant regions^20^. Additionally, other studies based on public surveys determined the sociodemographic factors affecting respondents’ behaviors after receiving the warning messages^21,22^. Another study determined the factors that improve the effectiveness of WEA by analyzing retweet data on Twitter^4^. However, studies statistically confirming the effectiveness of WEA during the COVID-19 pandemic remain insufficient.

Recently, researchers have analyzed public foot traffic data via mobile phone to understand the human mobility patterns or to identify the compliance of social distancing during the COVID-19 pandemic^23,24^. The results demonstrated that the lower level of public foot traffic was associated with the lower interpersonal contacts and the lower COVID-19 cases^11,25,26^.

## Results

### Correlation analysis

**Figure 2:**
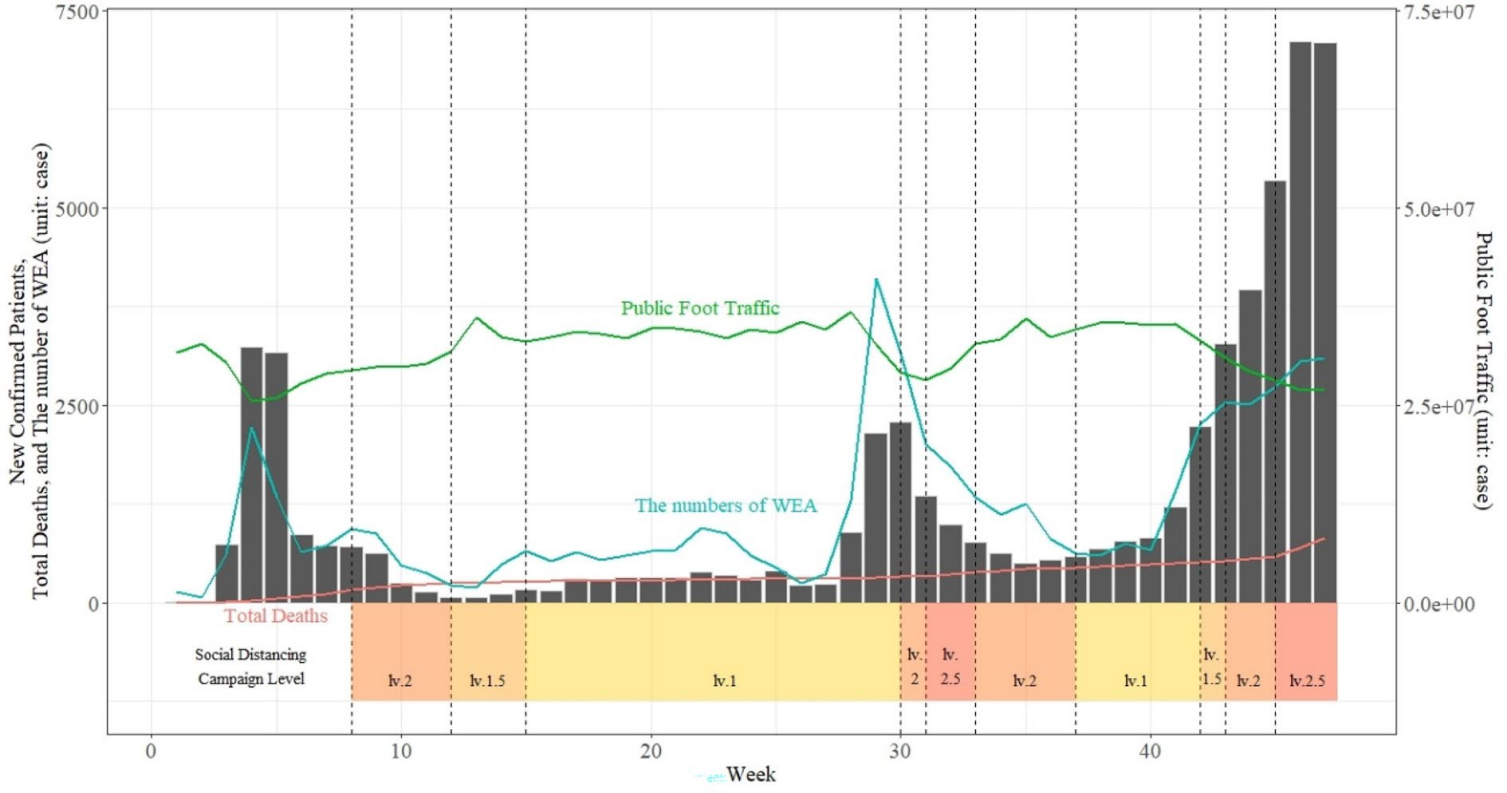
Overall confirmed cases of COVID-19 in Korea, 2020. The red line shows the cumulative death cases from COVID-19, the blue line shows the number of WEAs via mobile messages, and the green line indicates the public foot traffic. The social distancing campaign level is based on the cases of the national capital region (the boxes below the graph).

During the COVID-19 pandemic, more confirmed patients led to more WEA messages being issued (see Fig. 2). Table 1 shows the results of the correlation analysis of the nationwide cases. The number of confirmed COVID-19 cases and “warning” and “guidance” WEA messages were significantly correlated. The variables statistically significantly correlated with public foot traffic were “warning” messages, “guidance” messages, and COVID-19 confirmed cases.

**Table 1:** Results of the correlation analysis using the national data set.

Variables		[1]	[2]	[3]	[4]	[5]	[6]	[7]
WEA topics	“Warning” [1]	1						
	“Guidance” [2]	0.948**	1					
# of News [3]		0.251	0.188	1				
Confirmed	Cases [4]	0.828**	0.719**	0.252	1			
	Deaths [5]	0.286	0.231	0.662**	0.445**	1		
Social distancing campaign level [6]	Level [6]	0.330*	0.384**	0.281	0.212	0.379*	1	
	Public foot traffic [7]	− 0.584**	− 0.530**	0.007	− 0.480**	0.096	0.040	1

WEA, wireless emergency alerts.

*p < 0.05, **p < 0.01. Pearson Correlation Coefficient, N = 45.

**Figure 3:**
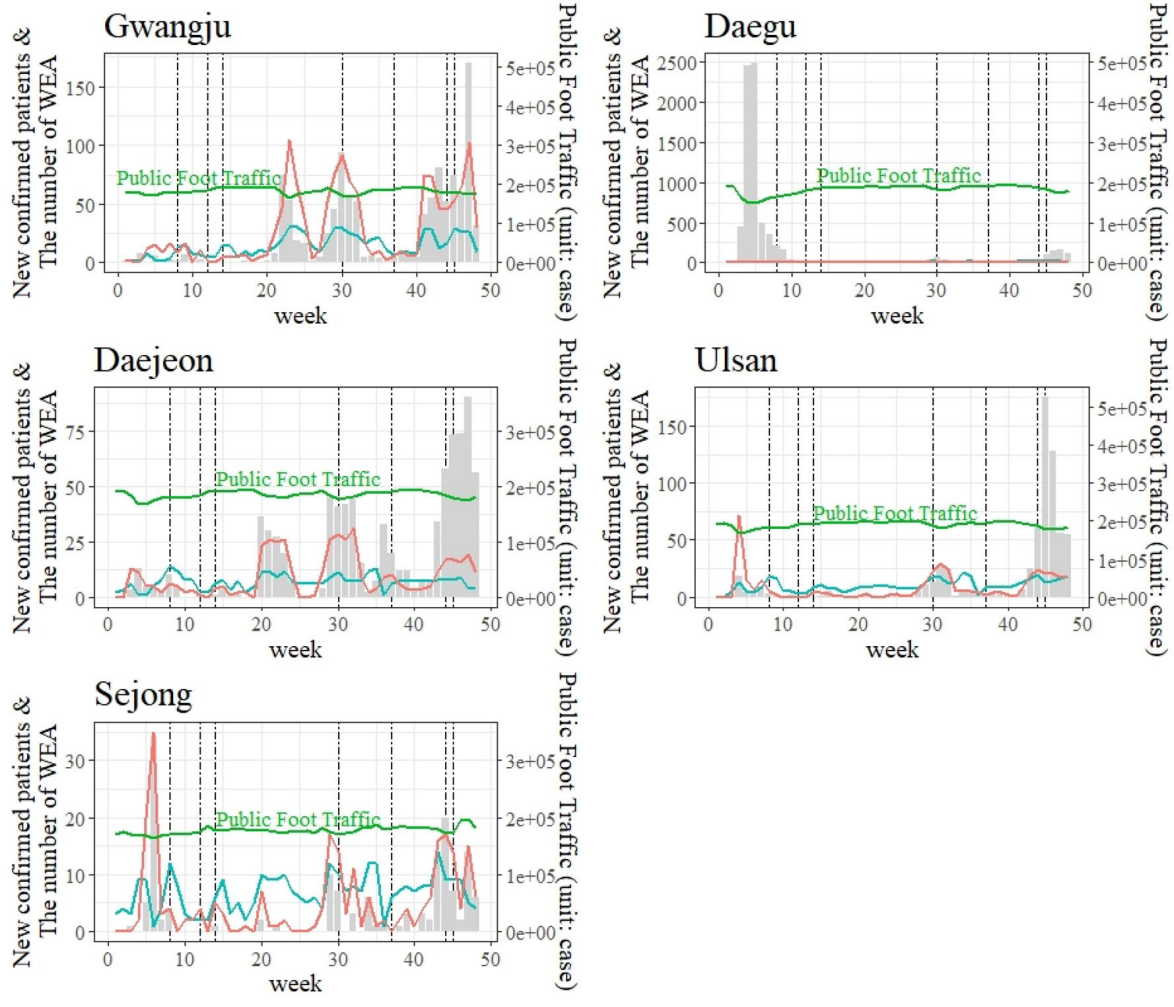
The weekly changes of confirmed COVID-19 cases and WEA messages in the target areas. The grey bar shows the number of weekly confirmed cases. The red line indicates the number of “warning” topic messages, the blue line is the “guidance” topic, and the green line shows the daily average public foot traffic.

As most WEA messages during COVID-19 were issued regionally by the local government, WEA messages and foot traffic data cannot be compared at the national level. The analysis needs to focus on the narrower areas of local governments—Gwangju, Daegu, Daejeon, Ulsan, and Sejong—to identify the effectiveness of WEAs on public foot traffic. Table 2 and Fig. 3 show the number of confirmed cases and WEA messages for each region.

**Table 2:** The number of confirmed cases and WEA messages by region.

City	Number of confirmed patients	Number of messages				
		Total	Sender^a^		Topic^b^	
			Local government	Central agency	“Warning”	“Guidance”
Gwangju	823	1534	1296	238	1130	633
Daegu	7192	475	237	238	89	430
Daejeon	628	697	459	238	403	313
Ulsan	435	698	460	238	337	431
Sejong	127	475	238	237	216	303

WEA, wireless emergency alerts.

^a^Local governments send messages to residents of their administrative districts. However, central agencies, such as the Korea Disease Control and Prevention Agency, generally send messages to the entire Korean population.

^b^Some messages contained both “warning” and “guidance” topics. Therefore, the sum of the “warning” and “guidance” topics is larger than the total number of messages.

The correlation analyses (Table 3) found that the public foot traffic of each city, excluding Daegu, was generally affected by the WEA messages. The “warning” and “guidance” topics were significantly highly correlated, except in Sejong, meaning that most local governments sent “warning” messages with “guidance” messages. The public foot traffic data negatively correlated with the number of “warning” topic messages and newly confirmed patients, except in Daegu. As the number of confirmed patients in Daegu increased considerably during the initial wave of COVID-19, Daegu city officials could not conduct the appropriate epidemiological investigation. Additionally, they could not send warning WEA messages to their residents.

**Table 3:** Results of the correlation analysis for five metropolitan cities.

City	Variables		[1]	[2]	[3]	[4]	[5]	[6]
Gwang-ju	WEA topics	“Waring” [1]	1					
		“Guidance” [2]	0.845**	1				
		# of news [3]	− 0.053	− 0.131	1			
		# of confirmed cases [4]	0.847**	0.682**	0.036	1		
		Social distancing level [5]	0.183	0.450**	0.227	0.248	1	
		Public foot traffic [6]	− 0.442**	− 0.269	0.061	− 0.534**	0.059	1
Daegu	WEA topics	“Waring” [1]	1					
		“Guidance” [2]	0.704**	1				
		# of news [3]	0.044	0.132	1			
		# of confirmed cases [4]	− 0.185	− 0.265	0.317*	1		
		Social distancing level [5]	0.206	0.566**	0.227	− 0.442**	1	
		Public foot traffic [6]	− 0.111	0.029	0.230	− 0.317*	0.298*	1
Daejeon	WEA Topics	“Waring” [1]	1					
		“Guidance” [2]	0.413**	1				
		# of news [3]	− 0.020	0.015	1			
		# of confirmed cases [4]	0.796**	0.287	0.044	1		
		Social distancing level [5]	0.212	0.456**	0.227	0.355*	1	
		Public foot traffic [6]	− 0.422**	− 0.086	0.128	− 0.412**	0.147	1
Ulsan	WEA Topics	“Waring” [1]	1					
		“Guidance” [2]	0.412**	1				
		# of news [3]	0.388**	0.260	1			
		# of confirmed cases [4]	0.398**	0.296*	0.094	1		
		Social distancing level [5]	− 0.055	0.495**	0.227	0.245	1	
		Public foot traffic [6]	− 0.638**	− 0.201	0.038	− 0.344*	0.061	1
Sejong	WEA Topics	“Waring” [1]	1					
		“Guidance” [2]	0.219	1				
		# of news [3]	0.395**	0.052	1			
		# of confirmed cases [4]	0.903**	0.125	0.290	1		
		Social distancing level [5]	− 0.101	0.217	0.227	− 0.120	1	
		Public foot traffic [6]	− 0.316*	− 0.071	0.045	− 0.326*	0.123	1

*p < 0.05, **p < 0.01. Pearson correlation coefficient, N = 45.

### Partial least squares path analysis

Ulsan is located in the southeastern part of the Korean Peninsula, where the number of confirmed cases peaked early. During the COVID-19 outbreak, Ulsan City Hall issued WEA messages to approximately 1.15 million citizens. Unlike the other cities, they decided that information about COVID-19 should be shared with all citizens in Ulsan, although new cases were confirmed in certain sub-districts^8^. The number of WEA messages issued by Ulsan City Hall was relatively large and had very few repeated contents^27^. Therefore, we selected Ulsan metropolitan city for a detailed analysis. Moreover, the patterns of the COVID-19 outbreak in Ulsan reflects that of the whole country, showing three waves of newly confirmed cases. This similarity means that Ulsan is the most appropriate place to validate the effectiveness of WEAs in Korea.

**Figure 4:**
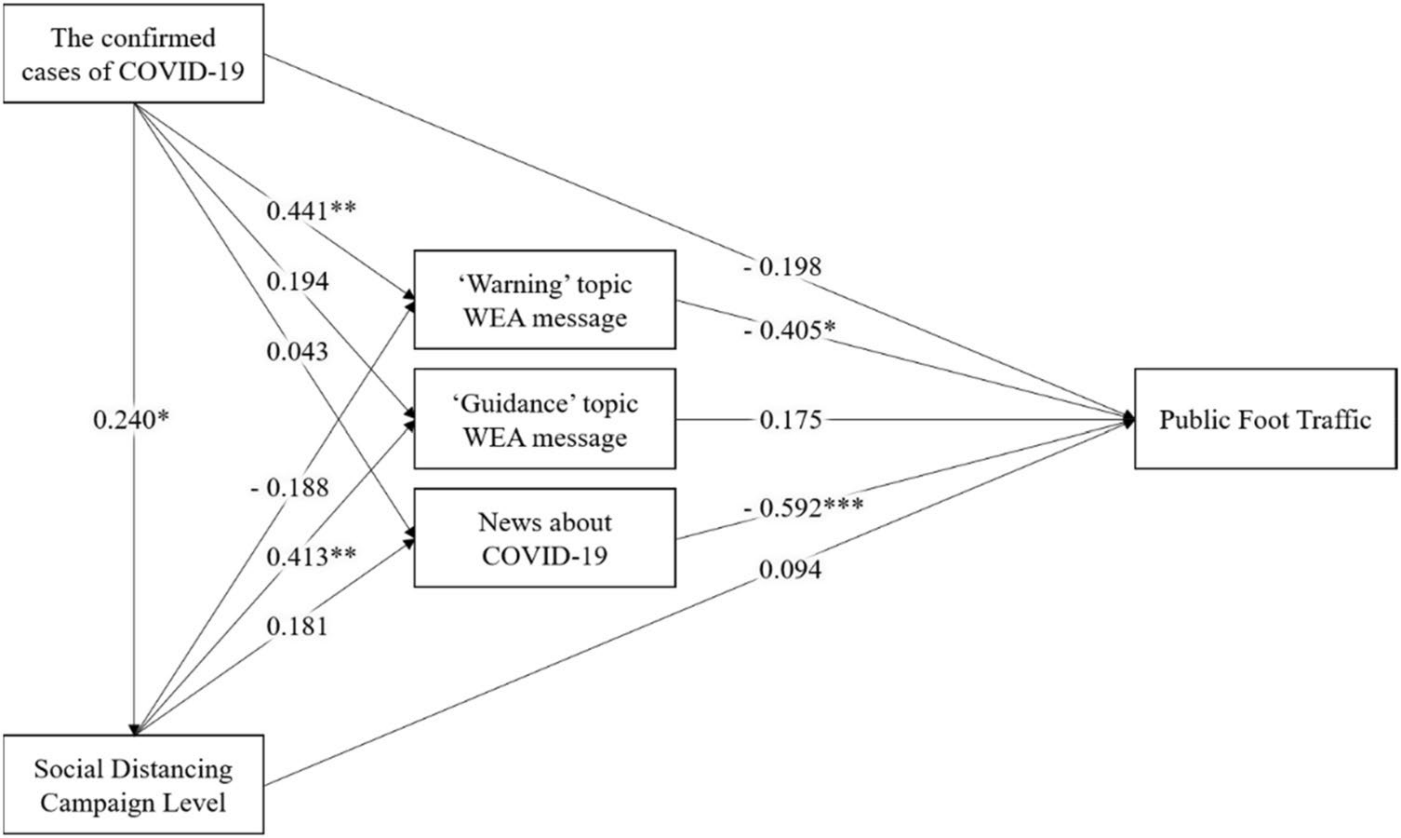
Partial least squares path model for Ulsan metropolitan city (*p < 0.05, **p < 0.01, ***p < 0.001).

Figure 4 shows the partial least squares (PLS) path analysis results for Ulsan Metropolitan City. The statistical significance of the structural paths in this model was identified using a bootstrap resampling technique that generated 500 bootstrap subsamples. Model fit was determined using four parameters. In Table 4, the two parameters (d_ULS, d_G) were within the acceptable level of less than 0.95, while the other fit indices were close to the recommended values^28,29^. This analysis identified the relationships between public foot traffic and the number of WEA messages and news media by assessing the ${R}^{2}$ value and path coefficient (β). The adjusted ${R}^{2}$ value of population movement was 0.69, which is higher than the acceptable level^30^.

**Table 4:** Results of overall model fit for the PLS path analysis.

Statistic	Recommended value	Obtained value
SRMR	< 0.08	0.135
d_ULS	< 0.95	**0.384**
d_G	< 0.95	**0.102**
NFI	> 0.9	0.790

SRMR, standardized root mean square residual; d_ULS, unweighted least squares discrepancy; d_G, geodesic discrepancy; NFI, normed fit index.

Significant values are in bold.

The path coefficient (β) was assessed at the significance level (see Table 5) to test the three hypotheses. The “warning” topic in WEA messages shows a statistically significant relationship with public foot traffic (β = − 0.405, p < 0.05), thus supporting H1. However, the “guidance” topic in WEA messages is not significantly related with public foot traffic (β = 0.175), thus rejecting H2. Finally, the relationship between news articles and public foot traffic was statistically significant (β = − 0.592, p < 0.001), thus supporting H3.

**Table 5:** Results of the path coefficients with t-value and p-value for the PLS-SEM.

Hypothesis	Path coefficient (β)	t-value	p-value
H1: “Warning” topic in WEA message → public foot traffic	− 0.405	2.128	0.034
H2: “Guidance” topic in WEA message → public foot traffic	0.175	1.285	0.199
H3: News about COVID-19 → public foot traffic	− 0.592	5.587	< 0.001

PLS-SEM, partial least squares structural equation modeling; WEA, wireless emergency alerts.

## Discussion

WEA emerged as an important platform for providing immediate disaster information during the COVID-19 pandemic; however, few studies have quantitatively analyzed the effectiveness of WEA messages. This study is meaningful, as the effectiveness of WEAs was quantitatively validated using public foot traffic data obtained from mobile big data. The results showed that the “warning” WEA topic was confirmed to be significantly correlated with reducing public foot traffic; that is, people followed social distancing measures, such as staying at home. The “warning” messages would have been recognized as important information because they included newly confirmed cases in their communities. However, the effect of the “guidance” WEA topic on foot traffic was not statistically significant. Guidance for preventive measures, such as wearing a face mask, was essential initially, but sending the same content repetitively reduced its effect.

Based on these results, emergency managers or public officials should consider that the notice about the relevant risks directly concerning the recipients is more effective than repeating the same guidance. They should prepare well-constructed message prototypes for immediately and rapidly issuing warnings in emergency situations^31^. People’s fatigue from receiving too many WEA messages has long been a social issue in Korea, as suggested in this study. Additionally, a controversy arose over a privacy infringement of disclosing route and patient information^32^. Consequently, the Ministry of the Interior and Safety—the WEA management agency—limited contents about general preventive measures, the entire routes of confirmed cases, unnecessary contents, and the prohibition of transmission at midnight.

This study had some limitations. First, we could not evaluate the long-term effects of WEA. This study analyzed 10 months of data starting from the outbreak of COVID-19 in 2020. However, as COVID-19 has prolonged and spread widely, people have grown more accustomed to it. Korea’s national COVID-19 alert level has remained “severe” for more than a year. Under this prolonged risk situation, people’s level of fatigue has also increased, thus decreasing WEA effectiveness. Therefore, the results of this study could not predict the long-term effects of WEAs. In particular, the data trend shows that the effectiveness of WEA messages gradually decreases over time. Therefore, research on long-term effect trend is needed.

Second, although the WEA messages were likely to impact the public foot traffic in all five cities, Ulsan was the only place suitable for quantitative analysis because of the different message sending policies of each city. Therefore, regional effects need to be tracked in more detail through more rigorous analysis to generalize the effects of WEA.

## Methods

### Data collection

This study uses five data sets: the WEA messages, news articles including the keyword “COVID-19,” confirmed COVID-19 cases in Korea, foot traffic data at the metropolitan level, and social distancing campaign level data. Data were collected from February 3 to December 13, 2020. All datasets were constructed weekly.

WEA messages were collected from the Ministry of the Interior and Safety (MOIS) database, the head of the managing institutions of WEAs. It contained 41,569 COVID-19-related messages sent in Korea. The WEA messages included the date and time they were sent, sending agencies, region, and content. The official issuance agencies were limited to the central ministries, agencies such as MOIS and Korea Disease Control and Prevention Agency (KDCA), and local governments in the Republic of Korea.

To identify the impact of WEA content on social distancing, we classified the messages into two categories. The first topic group contained the warning information (i.e., number of confirmed cases in the recipients’ local community and their travel information, such as visiting places and times). The other topic group contained the general protective measure guidance (i.e., recommendations for preventing infectious diseases, such as wearing a mask and washing hands with soap and running water)^31,33^.

To classify WEA messages, we performed pre-processing, word embedding, and classification. An open-source morpheme analyzer, Mecab-ko from KoNLPy (a Korean morpheme analysis Python package) was used to extract the nouns from the messages during pre-processing. In Korea, WEA messages typically display similar patterns because they are written based on the government’s WEA guidelines. In particular, messages are written in noun forms instead of verbs to convey more information concisely. Furthermore, in Korean, many verbs tend to be tokenized into nouns. For example, “recommend wearing masks” is tokenized into “mask” (noun), “wearing” (noun in Korean), and “recommending” (noun in Korean). Subsequently, the extracted words were vectorized using Word2Vec, a word-embedding tool published by Google^34^. A random forest (RF) model was applied to classify the vectorized messages into each topic. First, two authors manually labeled 800 randomly chosen message samples, and each tree in the RF was trained with this training data set. Next, the trained model predicted non-labeled messages (test set) by averaging the votes from its trees. Usually, classifier algorithms classify each document into one category; however, in the case of COVID-19-related WEA messages, some cases included multiple categories. For example, “The 90th to 92nd COVID-19 patients were found. Investigation in progress. Please follow quarantine rules, such as second-level social distancing and wearing masks (sender: Yongsan-gu, Seoul; date: August 25, 2020)” is manually classified as both “warning” and “guidance.” For these multi-label classification problems, we applied two single-label classification models to each category simultaneously^35^. In other words, we trained two RF models to predict whether a message is a “guidance” topic, a “warning” topic, or both.

The news articles from five major newspaper companies in Korea were collected by web crawling on Naver News, the largest media portal in Korea. The selected media were *Chosun Ilbo, JoongAng Ilbo, Donga Ilbo, Hankyoreh*, and *Kyunghyang Shinmun*, Korea’s most important newspaper companies. A total of 51,692 articles that included the keyword “COVID-19” were collected among 118,768 articles during the study period.

We used two datasets for the weekly confirmed COVID-19 cases by administrative districts (metropolitan level). First, the Ministry of Health and Welfare provided a dataset of cases in March. Second, we built a dataset of the cases before March by compiling the data reported from the KDCA and the websites of local governments.

The public foot traffic data is based on mobile data counting the inflow and outflow in a particular area provided by SKT, the largest mobile service company in Korea^36^. They measured the movements to visit other areas, except within their residential area, and to stay there for more than 30 min^37^. Foot traffic data during the spread of COVID-19 was jointly provided by Statistics Korea and SK Geovision, a big-data service platform of SKT. They analyzed the mobile data of SKT subscribers and estimated the movement of the entire population by applying weights for the region, gender, and age^37^. They provided nationwide weekly average foot traffic data and increased or decreased rate of weekly movement in the 17 metropolitan districts in Korea. We used differenced data to remove the dependence of the weekly foot traffic data on time.

The Korean government implemented a social distancing campaign. It included recommendations for the public (i.e., delay or cancelation of unnecessary meetings) and restrictions on business activities (i.e., 9 P.M. curfew for restaurants and cafes). The initial levels of social distancing were *enhanced social distancing* (from March 22 to April 19, 2020), *eased social distancing* (April 20 to May 5, 2020), and *distancing in daily life* (May 6 to August 22, 2020). Government policies were actively followed and effective in mitigating the spread. However, the initial levels were not systematized; therefore, the Korean government adjusted the levels on a more systematic five-level scheme—1 (least severe), 1.5, 2, 2.5, and 3 (most severe). This study regarded each initial level as a systematized scheme: *enhanced social distancing* as level 2, *eased social distancing* as level 1.5, and *distancing in daily life* as level 1. A second wave of the disease has occurred since August, with a major outbreak in the Seoul metropolitan region. Therefore, the government applied social distancing level 2 (August to October 2020) nationwide and level 2.5 (August to September 2020) and 2 (September 2020) in the Seoul area. Afterward, the level was adjusted with the third wave: level 1 (October to November 2020), 1.5 (December 2020), and 2 (from December 2020) nationwide and level 1.5 (November 2020), 2 (November to December 2020), and 2.5 (from December. 2020) in the Seoul region^2,37^.

### Statistical analysis

A correlation analysis was conducted for the national level and five metropolitan cities: Gwangju, Daegu, Daejeon, Ulsan, and Sejong. IBM SPSS Statistics 21.0 was used to identify relationships between the variables, especially the correlation of foot traffic with other variables. For a more detailed analysis of the Ulsan case, PLS path analysis was constructed using the Smart-PLS 3 software. The PLS path modeling, a sequence of regressions in terms of weight vectors^38^, can estimate the path models in case of a low sample size^39^. The model fit of the PLS path analysis was determined using the standardized root mean square residual, the geodesic discrepancy (d_G), the unweighted least squares discrepancy (d_ULS), and a normed fit index (NFI). The d_G and d_ULS are parameters used to quantify the discrepancy between two matrices^40^, and NFI is another model fit criterion used to compare the nested models within a sample^28,41^.
